# Chinese famine exposure in early life and metabolic obesity phenotype in middle age: Results from the China health and retirement longitudinal study

**DOI:** 10.3389/fendo.2022.975824

**Published:** 2022-09-20

**Authors:** Yunhan Xu, Qian Yi, Shiyi Shan, Jiali Zhou, Shuting Li, Leying Hou, Xinxin Ye, Jiayao Ying, Peige Song, Lin An

**Affiliations:** ^1^ Department of Maternal and Child Health, School of Public Health, Peking University, Beijing, China; ^2^ School of Public Health, Zhejiang University School of Medicine, Zhejiang University, Hangzhou, China; ^3^ Department of Sport and Exercise Science, College of Education, Zhejiang University, Hangzhou, China; ^4^ Women’s Hospital, Zhejiang University School of Medicine, Hangzhou, China

**Keywords:** famine, CHARLS, fetal, early childhood, metabolic obesity phenotype

## Abstract

**Objective:**

To assess the association between early life exposure to famine and the risk of metabolic obesity phenotypes among adults in middle age.

**Methods:**

The study selected two comparison groups. Comparison A consisted of a non-exposed group born between 1963-1965 from the China Health and Retirement Longitudinal Study (CHARLS) 2015 wave (N=862) and a fetal-exposed group born between 1959-1961 from the 2011 wave (N=507). Comparison B consisted of an early childhood-exposed group born between 1955-1957 from the 2011 wave (N=830) and a fetal-exposed group born between 1959-1961 from the 2015 wave (N=552). Multivariable logistic regressions were conducted to explore the associations between different periods of famine exposure and obesity, metabolic health status, and metabolic obesity phenotypes, with stratification by sex.

**Results:**

Compared with the non-exposed group, participants exposed to famine in the fetal period had a significantly lower risk of overweight/obesity (*OR*: 0.78, 95%*CI*: 0.63-0.97) and a higher risk of metabolically unhealthy status (*OR*: 1.73, 95%*CI*: 1.34-2.23) and metabolically unhealthy non-obesity (MUNO) (*OR*: 2.12, 95%*CI*: 1.46-3.08) at the age of 50-52 years. In the sex-stratified analysis, males exposed to famine in the fetal period had a significantly lower risk of overweight/obesity (*OR*: 0.59, 95%*CI*: 0.43-0.80) and metabolically healthy obesity (MHO) (*OR*: 0.56, 95%*CI*: 0.37-0.85), while such associations were not found in females. Compared with the early childhood exposure group, participants in the fetal exposure group had a significantly lower risk of metabolic unhealthy status (*OR*: 0.65, 95%*CI*: 0.51-0.85) and MUNO (*OR*: 0.50, 95%*CI*: 0.35-0.72). Those associations were observed in both males and females.

**Conclusion:**

Exposure to famine in early life increased the risk of metabolically unhealthy status in adulthood. Different metabolic subtypes should be identified at an early stage and followed by classification, intervention, and treatment.

## Introduction

The increasing prevalence of obesity in both developed and developing countries has become a significant international public health challenge (1). Since the 1970s, the global prevalence of adult obesity has nearly tripled (2). Studies have found that being overweight and obese might shorten life expectancy and increase mortality due to several chronic diseases such as cardiovascular disease (CVD), type 2 diabetes (T2DM), and some cancers (3–6). Obesity is often accompanied by a constellation of metabolic abnormalities, including insulin resistance, hyperglycemia, hypertension, and dyslipidemia (7). However, such metabolic abnormalities are not present for all obese individuals, and the term “metabolically healthy obesity (MHO)” refers to individuals who have excess weight but show no signs of metabolic disorders. Multiple studies have demonstrated that people with different obesity phenotypes, including metabolically unhealthy obesity (MUO), metabolically unhealthy non-obesity (MUNO), and MHO, have higher CVD risks than people with metabolically healthy non-obesity (MHNO) (8–10). Therefore, it is important to research on the range of obesity phenotypes in order to provide a scientific basis for personalized prevention strategies and contribute to the development of public health policies.

With the development and expansion of the developmental origins of health and disease (DOHaD) hypothesis over the previous decades, numerous epidemiologic studies have indicated that environmental factors in early life, such as nutrition, may affect the risk of non-communicable diseases in adulthood (11, 12). While research involving maternal nutritional interventions may raise ethical concerns and pose a pathogenic risk, major historical events such as the Dutch famine of 1944-1945 and the Chinese famine of 1959-1961 can allow for natural experiments and afford opportunities to study the long-term effects of malnutrition in early life. With the assumption that famine would lead to malnutrition in early life, many scholars have confirmed that people born during famine have a higher risk of chronic diseases in adulthood compared to those who have not experienced famine (13, 14).

Research based on famine events has found that early life exposure to famine could confer greater risks of developing obesity or T2DM in adulthood (15, 16). However, age issues may introduce bias and impair the conclusions of such studies, since most of these studies designate those born after the famine as the control and subjects born during the famine as the exposure group (17). Given that people in the control group must be younger than those in the exposure group, it is difficult to distinguish whether the increased risk is due to older age or malnutrition in early life. Aside from this limitation of previous research, no study has yet to explore the association between early life undernutrition and metabolic obesity phenotypes in middle age.

To fill these gaps, our study aimed to assess the association between early life exposure to famine and the risk of metabolic obesity phenotypes in middle age and reduce age bias as much as possible by using two waves of data in the China health and retirement longitudinal study (CHARLS). Additionally, our study aimed to explore the long-term effects of different famine exposure periods on metabolic obesity phenotypes stratified by sex.

## Methods

### Participants

Participants were sampled from the CHARLS, a nationally representative longitudinal survey of middle-aged and elderly adults of mainland China supported by Peking University. The goal of the CHARLS was to create a high-quality and representative micro-database of Chinese residents aged above 45 years and to enhance scientific research on aging (18). The CHARLS used a probability-proportional-to-size sampling strategy to collect information on a range of domains, from broad socioeconomic status to individual health status. The sample covers approximately 10,000 households and 17,500 individuals in 150 counties or districts and 450 residential communities or villages from 28 provinces. Details of the study design and sampling framework are available elsewhere (18). This national survey was first carried out in 2011 as the baseline and followed up every two years. Thus far, this nationwide survey has released five comprehensive datasets to the public, including the baseline 2011 wave, the follow up 2013, 2015, and 2018 waves, and the 2014 wave focusing on life history. Since only CHARLS 2011 and 2015 provided blood data for metabolic assessment, these two waves were selected for our study. The data collection was approved by the Biomedical Ethics Review Committee of Peking University and each participant has signed informed consent.

### Famine exposure and severity

**Figure 1:**
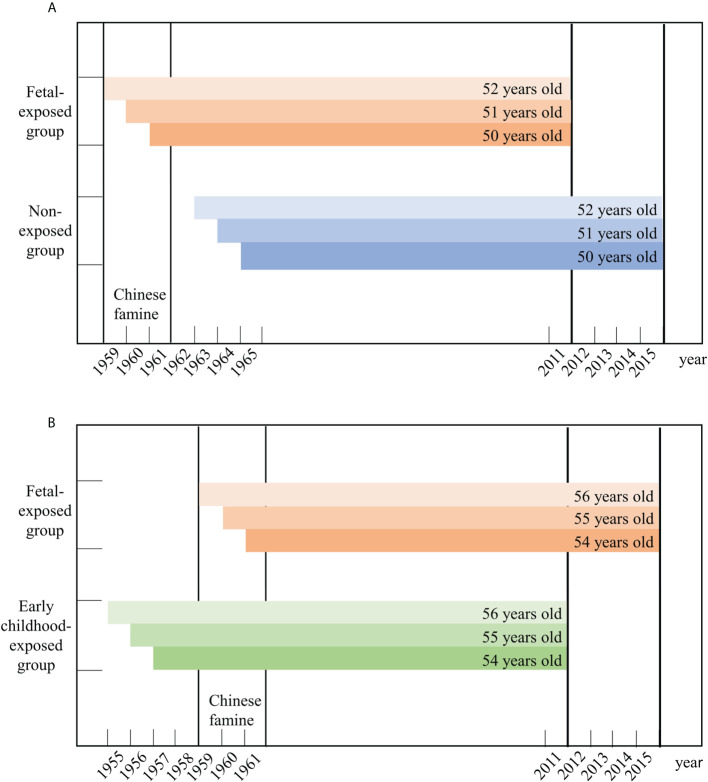
The selection of the participants in this study. **(A)** presents the selection of fetal-exposed group and non-exposed group aged 50-52 years old. **(B)** presents the selection of early childhood-exposed group and fetal-exposed group aged 54-56 years old

Since China experienced famine from 1959 to 1961, participants were classified into three exposure groups based on their birth dates: 1) the non-exposed group (born between 1 January 1963 and 31 December 1965); 2) the fetal-exposed group (born between 1 January 1959 and 31 December 1961); and 3) the early childhood-exposed group (born between 1 January 1955 and 31 December 1957) (19). To compare the health outcomes of groups, information from the non-exposed group from CHARLS 2015 and the fetal-exposed group from CHARLS 2011was used, so that the age of these two groups were both between 50 and 52 years. We also compared the health outcomes of the early childhood-exposed group from CHARLS 2011 and the fetal-exposed group from CHARLS 2015 based on a similar principle. **Figure 1** depicts a more detailed description of this process. Although the impact of the famine covered the entirety of mainland China, the severity of famine was varied by region due to differences in climate conditions, local policies, and population density (20). Referring to previous studies, the cohort size shrinkage indices (CSSI) from the 1% sample from the 2000 China Population Census was used as a proxy variable for the severity of the famine (21, 22). We measured famine severity by comparing average famine cohort size relative to non-famine cohort size in the population. The CSSI for this study was calculated as follows:


$$CSSI=\frac{N_{nonfamine}-N_{famine}}{N_{nonfamine}}$$

where N_famine_ is defined as the average cohort size of births during famine years (1959–1961) and N_nonfamine_ is the average cohort size of the births during the pre-famine years (1950–1957) (23). A higher CSSI indicates a greater reduction in the size of the famine cohorts due to the plunge of fertility or the surge of infant mortality and more severely affected by famine. Famine severity was assessed by CSSI at the provincial level, which is listed in **Table S1**.

### Definition of metabolic obesity phenotype

Using diagnostic criteria from the Working Group on Obesity in China (WGOC), participants were divided into either the non-overweight/obesity group (body mass index, BMI<24kg/m^2^) or the overweight/obesity group (BMI ≥24 kg/m^2^) (24). According to the modified National Cholesterol Rationale Education Program Adult Treatment Program III (NECP-ATP III), participants who met greater or equal to two of the following four criteria were defined as metabolically unhealthy: (1) triglyceride (TG) levels ≥150 mg/dL or use of lipid-lowering drugs: (2) high-density lipoprotein cholesterol (HDL-C) levels<40mg/dL for men or<50 mg/dL for women: (3) fasting plasma glucose (FPG) ≥100 mg/dL or drug treatment for elevated glucose: (3) systolic blood pressure (SBP) ≥130 mmHg or diastolic blood pressure (DBP) ≥85 mmHg or drug treatment for hypertension (25, 26).

Based on BMI classification and metabolic health status, participants were categorized into four groups: 1) MHNO; 2) MUNO; 3) MHO; and 4) MUO.

### Covariates

Covariates were divided into three areas: demographic characteristics, socio-economic factors, and health behaviors. Demographic characteristics included sex and marital status (unmarried, married); socio-economic factors included the highest education level (primary school or below, junior school, senior school or above) and region (urban, rural); health behaviors included smoking status and drinking status. Participants who smoked ≥100 cigarettes in their lifetime were identified as smokers and the rest as non-smokers. Participants were classified as drinkers if they drank at least once per week in the last year and the rest were considered non-drinkers. Participants who had heart problem (heart attack, coronary heart disease, angina, congestive heart failure, or other heart problems) or stroke were classified into the participants with the history of CVD.

### Statistical analysis

Categorical variables were reported using percentages (%) and the differences between groups were analyzed by chi-square (χ²) tests. Continuous variables that fit a normal distribution were reported as the mean ± SD and were analyzed by *t*-tests between groups. Continuous variables that were not normally distributed were described using the median (1st quartile, 3rd quartile) and a Wilcoxon rank sum test was adopted to compare between groups.

Multivariable logistic regressions were used to explore the associations between famine exposure and obesity, metabolic health status or metabolic obesity phenotypes. Crude model did not adjust for any covariates. Fully adjusted model was adjusted for age, sex, marital status, education, region, smoking, drinking status, CVD history and famine severity. Considering that the impact of famine might differ between males and females, a sex-stratified analysis was further conducted.

Statistical analyses were conducted in SAS, Version 9.4. A two-sided *p*-value of less than 0.05 was considered significant.

## Results

The study initially chose participants who were born between 1955-1957 and 1959-1961 from CHARLS 2011 (17,708) and born between 1959-1961 and 1963-1965 from CHARLS 2015 (21,100). After excluding participants with missing or invalid data, the fetal-exposed group from CHARLS 2011 (n=504) and the non-exposed group from CHARLS 2015 (n=833) (Comparison A), and the fetal-exposed group from CHARLS 2015 (n=527) and the early childhood-exposed group from CHARLS 2011 (n=820) (Comparison B) were included in the analysis (**Figure S1**) **Table S2** shows demographic characteristics of included and excluded participants.

The basic characteristics of participants according to famine exposure are shown in **Table 1**. For Comparison A, both the non-exposed group and fetal-exposed group were aged 50-52 years. No significant differences were found in sex, age, marital status, region and smoking status between the two groups, but a significant difference was found in education level and drinking status. Compared with the non-exposed group, people exposed to famine in the fetal period were more likely to have higher SBP and FPG levels, and lower HDL-C levels (*P*<0.05). Significant differences were also found in the prevalence of metabolically unhealthy status, obesity and different metabolic obesity phenotypes. The proportions of MHNO, MUNO, MHO and MUO in the participants not exposed to famine during early life were 31.0%, 11.9%, 22.2% and 34.9%, respectively. The proportions of MHNO, MUNO, MHO and MUO in participants exposed to famine in the fetal period were 27.8%, 21.2%, 14.1% and 36.9%, respectively.

**Table 1:** Basic characteristics of participants according to famine exposure.

Characteristic	*Comparison A*			*Comparison B*		
	Non-exposed	Fetal-exposed	*P*-value	Early childhood-exposed	Fetal-exposed	*P*-value
Total, n	833	504		820	527	
Sex, n (%)			0.280			0.815
Male	501 (60.1)	288 (57.1)		514 (62.7)	327 (62.0)	
Female	332 (39.9)	216 (42.9)		306 (37.3)	200 (38.0)	
Age, years	51.13 ± 0.85	51.12 ± 0.83	0.929	55.03 ± 0.86	55.10 ± 0.86	0.171
Marital status, n (%)			0.160			0.413
Unmarried	27 (3.2)	24 (4.8)		47 (5.7)	36 (6.8)	
Married	806 (96.8)	480 (95.2)		773 (94.3)	491 (93.2)	
Education, n (%)			< 0.001			< 0.001
Primary school or below	371 (44.6)	231 (45.8)		493 (60.1)	232 (44.0)	
Junior school	326 (39.1)	147 (29.2)		195 (23.8)	161 (30.6)	
Senior school or above	136 (16.3)	126 (25.0)		132 (16.1)	134 (25.4)	
Region, n (%)			0.284			0.576
Urban	337 (40.5)	189 (37.5)		305 (37.2)	204 (38.7)	
Rural	496 (59.5)	315 (62.5)		515 (62.8)	323 (61.3)	
Smoking, n (%)			0.525			0.977
Non-smoker	428 (51.4)	268 (53.2)		379 (46.2)	244(46.3)	
Smoker	405 (48.6)	236 (46.8)		441 (53.8)	283 (53.7)	
Drinking, n (%)			0.037			0.735
Non-drinker	479 (57.5)	319 (63.3)		495 (60.4)	323 (61.3)	
Drinker	354 (42.5)	185 (36.7)		325 (39.6)	204 (38.7)	
CVD history, n (%)			0.790			0.194
Yes	66 (7.9)	42 (8.3)		98 (11.9)	51 (9.7)	
No	767(92.1)	462 (91.7)		722 (88.1)	476 (90.3)	
SBP, mmHg	124.15 ± 17.66	128.04 ± 19.28	< 0.001	128.95 ± 20.26	127.50 ± 18.52	0.100
DBP, mmHg	77.49 ± 12.22	78.34 ± 12.62	0.227	77.61 ± 12.88	78.16 ± 11.8	0.425
TG, mg/dL	168.63 ± 106.20	174.35 ± 171.24	0.499	148.12 ± 114.33	163.87 ± 1104.50	0.009
HDL-C, mg/dL	46.54 ± 9.31	45.03 ± 13.73	0.030	46.78 ± 14.20	47.62 ± 10.97	0.225
FPG, mg/dL	99.23 ± 30.12	112.50 ± 38.97	< 0.001	110.67 ± 36.21	101.57 ± 29.17	< 0.001
Metabolic status, n (%)			< 0.001			0.007
Metabolically healthy	443 (53.2)	211 (41.9)		366 (44.6)	275 (52.2)	
Metabolically unhealthy	390 (46.8)	293 (58.1)		454 (55.4)	252 (47.8)	
Obesity, n (%)			0.029			0.017
Normal weight	357 (42.9)	247 (49.0)		439 (53.5)	247(46.9)	
Overweight/obesity	476 (57.1)	257 (51.0)		381 (46.5)	280 (53.1)	
Metabolic obesity phenotype, n (%)			< 0.001			< 0.001
MHNO	258 (31.0)	140 (27.8)		253 (30.8)	175(33.2)	
MUNO	99 (11.9)	107 (21.2)		186 (22.7)	72 (13.7)	
MHO	185 (22.2)	71 (14.1)		113 (13.8)	100 (19.0)	
MUO	291 (34.9)	186 (36.9)		268 (32.7)	180 (34.1)	

Data were presented as n (%) or means ± standard deviation (SD).

P-value represented T-test for continuous variables or χ2-test for categorical variables.

Comparison A represented the comparison between the fetal-exposed group and the non-exposed group aged 50-52 years; Comparison B represented the comparison between the early childhood-exposed group and the fetal-exposed group aged 54-56 years.

CVD, cardiovascular disease; SBP, systolic blood pressure; DBP, diastolic blood pressure; TG, triglyceride; HDL-C, high-density lipoprotein cholesterol; FPG, fasting plasma glucose; MHNO, metabolically healthy non-obesity; MUNO, metabolically unhealthy non-obesity; MHO, metabolically healthy obesity; MUO, metabolically unhealthy obesity.

For Comparison B, participants from the two groups were aged 54-56 years. There were no significant differences observed in sex, age, marital status, region, smoking and drinking status between the two groups, but there was a significant difference in education level between the two groups. Compared with the early childhood-exposed group, people exposed to famine in the fetal period were more likely to have higher TG levels and lower FPG levels (*P*<0.05). Significant differences between the two groups were also found in the prevalence of metabolically unhealthy status, obesity and different metabolic obesity phenotypes. The proportions of MHNO, MUNO, MHO and MUO in participants exposed to famine in early childhood were 30.8%, 22.7%, 13.8% and 32.7%, respectively. The proportions of MHNO, MUNO, MHO and MUO in participants exposed to famine in the fetal period were 33.2%, 13.7%, 19.0% and 34.1%, respectively.

**Table 2** shows the associations of the fetal-exposed group with metabolic status, obesity and metabolic obesity phenotypes compared with the non-exposed group aged 50-52 years. Compared with non-exposed participants, participants in the fetal-exposed group had a significantly higher risk of metabolically unhealthy status (Crude model: *OR_c_*: 1.58, 95%*CI*: 1.26-1.97) and a lower risk of obesity/overweight (Crude model: *OR_c_*: 0.78, 95%*CI*: 0.63-0.97). After adjusting for covariates, the associations were both strengthened (metabolically unhealthy, fully adjusted model: *OR_a_*: 1.73, 95%*CI*: 1.34-2.23; obesity/overweight, fully adjusted model: *OR_a_*: 0.75, 95%*CI*: 0.59-0.94, details in **Table 2**). The higher risk of metabolically unhealthy status in the fetal-exposed group was observed in both males (Fully adjusted model: *OR_a_*: 1.51, 95%*CI*: 1.10-2.07) and females (Fully adjusted model: *OR_a_*: 2.24, 95%*CI*: 1.44-3.50) after adjusting for all covariates. Moreover, males in the fetal-exposed group still had a significantly lower risk of obesity/overweight compared with non-exposed males (Fully adjusted model: *OR_a_*: 0.59, 95%*CI*: 0.43-0.80), whereas there was no difference in obesity/overweight risk for females.

**Table 2:** The associations of the fetal-exposed group with metabolic status, obesity and metabolic obesity phenotypes compared with the non-exposed group [*OR* (95%*CI*)].

Characteristics	Metabolic status		Obesity		Metabolic obesity phenotype			
	MH	MU	NO	Overweight/obesity	MHNO	MUNO	MHO	MUO
Overall								
Crude model	1	1.58 (1.26,1.97)	1	0.78 (0.63,0.97)	1	1.99 (1.42,2.80)	0.71 (0.50,1.00)	1.18 (0.89,1.55)
Fully adjusted model	1	1.73 (1.34,2.23)	1	0.75 (0.59,0.94)	1	2.12 (1.46,3.08)	0.69 (0.48,0.98)	1.25 (0.92,1.71)
Male								
Crude model	1	1.39 (1.03,1.88)	1	0.62 (0.46,0.83)	1	2.20 (1.35,3.60)	0.60 (0.40,0.88)	0.87 (0.60,1.27)
Fully adjusted model	1	1.51 (1.10,2.07)	1	0.59 (0.43,0.80)	1	2.42 (1.45,4.03)	0.56 (0.37,0.85)	0.89 (0.60,1.33)
Female								
Crude model	1	2.19 (1.43,3.35)	1	1.02 (0.71,1.47)	1	3.31 (1.69,6.49)	1.55 (0.72,3.34)	2.54 (1.37,4.72)
Fully adjusted model	1	2.24 (1.44,3.50)	1	1.02(0.70,1.48)	1	3.41 (1.69,6.85)	1.54(0.69,3.44)	2.66 (1.40,5.07)

OR, odds ratio; CI, confidence interval.

Crude model did not adjust for any covariate. Fully adjusted model was adjusted for age, gender (except for sex-stratified analyses), marital status, education, region, smoking, drinking status, CVD history and CSSI.

MH, metabolically healthy; MU, metabolically unhealthy; NO, non-overweight/obesity; MHNO, metabolically healthy non-obesity; MUNO, metabolically unhealthy non-obesity; MHO, metabolically healthy obesity; MUO, metabolically unhealthy obesity.

Compared with the risk of MHNO, participants in the fetal-exposed group had a higher risk of MUNO than those in the non-exposed group before adjusting for covariates (Crude model: *OR_c_*: 1.99, 95%*CI*: 1.42-2.80). That association was strengthened after adjusting for covariates (Fully adjusted model: *OR_a_*: 2.16, 95%*CI*: 1.51-3.11). And a weak association was between fetal exposure to famine and the risk of MHO (Fully adjusted model: *OR_a_*: 0.69, 95%*CI*: 0.48-0.98). No significant association between fetal exposure to famine and the risk of MUO was found when compared with not exposed to famine. In the sex-stratified analysis, after adjusting for all covariates, compared with the risk of MHNO, males in the fetal-exposed group had a higher risk of MUNO (Fully adjusted model: *OR_a_*: 2.42, 95%*CI*: 1.45-4.03) and a lower risk of MHO (Fully adjusted model: *OR_a_*: 0.56, 95%*CI*: 0.37-0.85) compared with non-exposed participants. However, females exposed to famine during the fetal period showed increased risks to both MUNO (Fully adjusted model: *OR_a_*: 3.41, 95%*CI*: 1.69-6.85) and MUO (Fully adjusted model: *OR_a_*: 2.66, 95%*CI*: 1.40-5.07). Moreover, no difference was observed in the risk of MHO between fetal-exposed and non-exposed females.

The associations of the fetal-exposed group with metabolic status, obesity and metabolic obesity phenotypes compared with the early childhood-exposed group aged 54-56 years are shown in **Table 3**. Compared with early childhood-exposed participants, participants in the fetal-exposed group had a significantly lower risk of metabolically unhealthy status (Crude model: *OR_c_*: 0.74, 95%*CI*: 0.60-0.92). After adjusting for covariates, this association was strengthened (metabolically unhealthy, fully adjusted model: *OR_a_*: 0.65, 95%*CI*: 0.51-0.85, details in **Table 3**). Notably, a lower risk of metabolically unhealthy status in the fetal-exposed group was observed both in males (Fully adjusted model: *OR_a_*: 0.69, 95%*CI*: 0.51-0.94) and females (Fully adjusted model: *OR_a_*: 0.57, 95%*CI*: 0.36-0.91) after adjusting for all covariates. Nevertheless, participants in the fetal-exposed group had a significantly higher risk of overweight/obesity (Crude model: *OR_c_*: 1.31, 95%*CI*: 1.05-1.63) compared with early childhood-exposed participants, but the association was not significant after adjusting for all covariates. Despite the fact that females in the fetal-exposed group had a significantly higher risk of overweight/obesity in the crude model (Crude model: *OR_c_*: 1.50, 95%*CI*: 1.03-2.18), there was no difference in overweight/obesity risk for both males and females between these two groups adjusting for covariates.

**Table 3:** The associations of the fetal-exposed group with metabolic status, obesity and metabolic obesity phenotypes compared with the early childhood-exposed group [*OR* (95%*CI*)].

Characteristics	Metabolic status		Obesity		Metabolic obesity phenotype			
	MH	MU	NO	Overweight/obesity	MHNO	MUNO	MHO	MUO
Overall								
Crude model	1	0.74 (0.60,0.92)	1	1.31 (1.05,1.63)	1	0.56 (0.40,0.78)	1.28 (0.92,1.78)	0.97 (0.74,1.27)
Fully adjusted model	1	0.65 (0.51,0.85)	1	1.26 (0.99,1.59)	1	0.50 (0.35,0.72)	1.17 (0.83,1.66)	0.84 (0.62,1.15)
Male								
Crude model	1	0.71 (0.53,0.95)	1	1.22 (0.92,1.61)	1	0.57 (0.36,0.89)	1.26 (0.88,1.82)	0.91 (0.63,1.32)
Fully adjusted model	1	0.69 (0.51,0.94)	1	1.15 (0.86,1.55)	1	0.55 (0.35,0.89)	1.16 (0.79,1.70)	0.87 (0.59,1.28)
Female								
Crude model	1	0.60 (0.39,0.94)	1	1.50 (1.03,2.18)	1	0.44 (0.23,0.84)	1.23 (0.55,2.74)	0.81 (0.45,1.43)
Fully adjusted model	1	0.57 (0.36,0.91)	1	1.45 (0.99,2.15)	1	0.42 (0.22,0.82)	1.13 (0.50,2.59)	0.74 (0.40,1.36)

OR, odds ratio; CI, confidence interval.

Crude model did not adjust for any covariate. Fully adjusted model was adjusted for age, gender (except for sex-stratified analyses), marital status, education, region, smoking, drinking status, CVD history and CSSI.

MH, metabolically healthy; MU, metabolically unhealthy; NO, non-overweight/obesity; MHNO, metabolically healthy non-obesity; MUNO, metabolically unhealthy non-obesity; MHO, metabolically healthy obesity; MUO, metabolically unhealthy obesity.

As compared with the risk of MHNO, participants in the fetal-exposed group had a lower risk of MUNO than those in the early childhood-exposed group before adjusting for covariates (Crude model: *OR_c_*: 0.56, 95%*CI*: 0.40-0.78). Additionally, this association was strengthened after adjusting for covariates (Fully adjusted model: *OR_a_*: 0.50, 95%*CI*: 0.35-0.72). No significant associations in participants aged 54-56 years were found between fetal exposure to famine and the risks of MHO and MUO when compared with early childhood exposure to famine. In the sex-stratified analysis, after adjusting for all covariates, both males (Fully adjusted model: *OR_a_*: 0.55, 95%*CI*: 0.35-0.89) and females (Fully adjusted model: *OR_a_*: 0.42, 95%*CI*: 0.22-0.82) in the fetal-exposed group had a lower risk of MUNO compared to those in the early childhood-exposed group compared with the risk of MHNO For the sensitivity analysis, **Table S3** shows the associations of the fetal-exposed group with metabolic status, obesity and metabolic obesity phenotypes compared with the age-balanced group..

## Discussion

In the first section of our study (Comparison A), the fetal-exposed group in CHARLS 2011 was compared with the non-exposed group in CHARLS 2015, and it was found that fetal famine exposure in early life led to lower risks of overweight and obesity. However, in the sex-stratified analysis, this significant association was only observed in males. No such association was observed in females, regardless of whether the covariates were controlled or not. In the second section of our study (Comparison B), no significant difference was observed with regard to risk of overweight and obesity in the fetal exposure group in CHARLS 2015 compared with the early childhood exposure group in CHARLS 2011. Similar results were obtained in the sex-stratified analysis.

The results of our study align with other published studies that explored the effects of famine exposure on obesity in adulthood. As early as 1976, one study based on Dutch famine indicated that during the last trimester of pregnancy and the first months of life, exposure to famine resulted in lower obesity rates in young males (27). For Chinese famine, Wang N et al. also used CHARLS 2011 baseline wave data to analyze the association between famine exposure and BMI, overweight, and central obesity by the difference-in-differences (DID) approach, and they found that famine exposure in the fetal period or childhood was associated with decreased BMI. Additionally, their study concluded that famine exposure might have health-promoting effects through reduced BMI and risks of overweight and abdominal obesity in middle age (28).

In contrast, the results of our study are inconsistent with other studies in some ways. For instance, one study conducted in Qingdao found that famine exposure in early life could increase the risk of obesity in adulthood based on two population-based surveys of 8185 subjects (29). Furthermore, a systematic review and meta-analysis of 20 related studies showed that exposure to famine during early life might increase the risk of elevated BMI and obesity, especially in women, people with fetal famine exposure, and people under the age of 50 years (30). There are several reasons why the results of our study may have differences from previous studies: First, the differences may be attributed to differences in study design, the definition of famine exposure, study subjects, and regions of study. The two cross-sectional surveys used to control the confounding factor of age might be the main reason for the inconsistent results between the current and previous studies. According to their reports, some studies such as the study by Liu et al. directly compared the fetal/infant group (born between 01/01/1959 and 12/31/1961) with the non-exposed group (born between 01/01/1962 and 12/31/1971) without considering uncontrolled age differences between two groups and the varying risk of overweight and obesity with age. In the same way, most of the studies included in the meta-analysis above did not consider the effect of age. As a result, the positive association between famine exposure and the risk of overweight and obesity might be overestimated, which accords with a previous study from Jiang et al. based on the Chinese Health and Nutrition Survey (CHNS) (31). The study by Jiang et al. indicated that fetal exposure to the famine in females was associated with higher risks of overweight and central obesity before adjusting for age, but that after the adjustment, the association between fetal exposure to the famine and central obesity disappeared and the risk of central obesity even decreased. Meanwhile, a similar transformation was also observed in males. The study further demonstrated the necessity of controlling age factors and suggested that the effects of famine exposure in early life were weak and easily masked by confounding factors. Secondly, the natural event of the Chinese Famine had a certain mortality selection effect, which refers to the possibility that people who were exposed to famine in early life but survived tended to be unusually well endowed with some genetic trait that might reduce the risk of chronic disease in adulthood (32). When the effect of mortality selection outweighs the effect of famine-induced debilitation, the risk of obesity in adulthood may decrease. Thirdly, publication bias and limited sample size may have led to inconsistent results.

Beyond these inconsistencies with past studies, one noteworthy finding from this study was the sex differences in the risk of overweight and obesity between the non-exposed and fetal-exposed groups. A retrospective cohort study based in Guangdong Province analyzed the physical indicators of 12,065 subjects born between 1957 and 1964 and found that males born during famine years had a lower risk of obesity in middle age, but there was no such association among females, which was consistent with the results of our study (33). Furthermore, another study indicated that an increased risk of obesity was only observed in females and another study observed that the association between famine exposure and increased risk of obesity was stronger in females than in males (34, 35). These findings suggest females exposed to famine in early life might have an increased risk of obesity in adulthood, while males’ risk of obesity tended to remain unchanged or even decrease moderately. The mechanism behind this is not clear, but one study suggested that famine exposure during pregnancy had an impact on the sex ratio of live births, with boys having a lower proportion of live births (36). Therefore, the sex difference in the risk of obesity may be related to the fact that males were more sensitive to the external environment in early life, suffered a relatively stronger selection effect, and had higher mortality (37). Additionally, several studies indicated that famine exposure in early life might be related to a younger age at menopause (38, 39). This could lead to a loss of the protective effect of estrogen at an earlier age in females who experienced famine in early life, and might have an increased risk of obesity (40). It is possible that the combination of this effect and the effect of debilitation may balance the mortality selection effect. However, more research is needed in this area.

The first section of our study (Comparison A) suggested that both males and females who were exposed to famine during the fetal period had a significantly higher risk of being metabolically unhealthy at the age of 50 to 52 years compared with the non-exposed group. This is in contrast to the second section of our study (Comparison B), which found that males and females exposed to famine during the fetal period had a lower risk of metabolically unhealthy status at the age of 54 to 56 years compared to those in the early childhood-exposed group after adjusting for all covariates.

Consistent with the results of most studies related to the Chinese Famine, our study suggests that exposure to famine in early life is associated with an increased risk of metabolically unhealthy status in middle age. For example, one other study concluded that exposure to famine during the fetal period was associated with an increased risk of metabolic syndrome in adulthood (41). One meta-analysis also showed that people born during the famine had an increased risk of metabolic syndrome in adulthood compared with people in the control group born either after or before the famine (14).

In contrast, the results of our study were not consistent with the study of Wang et al. Using the data from CHARLS 2011, they found that infant famine exposure significantly increased the risk of metabolic syndrome in adulthood, but similar results were not observed in the fetal-exposed group (42). The different findings between our study and the one conducted by Wang et al. could be attributed to different criteria for judging metabolic health status. For instance, the study conducted by Wang et al. used the diagnostic criteria recommended by Chinese Diabetes Society, while our study used criteria from NCEP-ATP III. In addition to the study from Wang et al., studies on the Dutch Famine have also not found a link between famine exposure in early life and metabolic syndrome in adulthood, which may be related to the severity and duration of famine in different regions (43). After all, the Dutch famine lasted only six months and the food supply was quickly restored within three weeks of the end; whereas the Chinese famine lasted for three years and covered a wide range of areas, which may have permanently changed the structure and function of the body among those impacted (44).

In the first section of our study (Comparison A), it was found that compared with the risk of MHNO, males in the fetal-exposed group had a higher risk of MUNO and lower risk of MHO than those in the non-exposed group. Moreover, fetal exposure to famine among females showed increased risks of both MUNO and MUO and there was no difference observed in the risk of MHO between fetal-exposed and non-exposed females. The second section of our study (Comparison B) showed that, taking MHNO as a reference, both males and females exposed to famine during the fetal period had a lower risk of MUNO than those in the early-childhood exposed group after adjusting for all covariates.

Our study indicated that early life exposure to famine led to an increased risk of MUNO regardless of sex. The simplified results of the whole study also suggest that exposure to famine in early life might be a protective factor for obesity and a risk factor for metabolically unhealthy status. A study based on the Beijing Child and Adolescent Metabolic Syndrome Study (BCAMS) found that low birth weight was an independent risk factor for MUNO phenotype, which is consistent with the results of our study, because birth weight is a reflection of the intrauterine nutritional environment and a good measure of nutritional status in early life (45–47). A few studies have suggested that an adverse intrauterine environment could permanently affect the function of metabolism-related tissues through epigenetic modifications, thus increasing the risk of metabolic abnormalities in adulthood (48, 49). Famine exposure in early life may affect the development of MUNO in adulthood by affecting the function and distribution of metabolic tissues such as fat. The cohort study also showed that people with high birth weight (>75th percentile) who subsequently assumed a normal weight had a lower risk of becoming metabolically unhealthy, suggesting the importance of nutrition in early life (47).

Compared with MHO and MUO, people with the MUNO phenotype are often ignored because of their normal body size, but in fact, the risks of insulin resistance, chronic kidney disease, and long-term cardiovascular and cerebrovascular diseases in this phenotype of people are higher than those in the MHO population who are metabolically healthy but obese (50–52). Furthermore, it is noteworthy that a study including 1950 normal weight subjects of different ethnic groups in different countries found that the prevalence of MUNO phenotype was significantly higher in Asians than in Caucasians (53). Therefore, in normal weight people, especially Asian people who were exposed to the famine during their fetal period and childhood, it is more significant to identify different metabolic subtypes in the early stages and carry out interventions and treatment specific to different phenotypes to achieve accurate prevention of metabolic abnormalities and rational allocation of medical resources to prevent chronic diseases.

The present study had the following strengths. First, our study was the first to explore the associations between famine exposure in early life and metabolic obesity phenotypes. In addition, the results provided the scientific basis for personalized prevention strategies for middle-aged and elderly people born on specific dates. Second, our study creatively selected participants from two cross-sectional surveys, the CHARLS 2011 and 2015, at the same age period, which made the outcomes of groups more comparable and reduced the effects of unadjusted age, which can lead to bias in other conventional study designs. The appropriate analytical method used in our study controlled for age difference between comparison groups due to non-overlapping date of birth. Third, our study assessed regional famine intensity using CSSI derived from population census data, which enjoyed popularity in different disciplines and constructed multiple levels of famine exposure. Fourth, the results of both crude and adjusted models were presented and could lead to further examination on primary study results. Lastly, compared to other famines like the Dutch famine of 1944-1945, the Great Chinese Famine lasted longer, covered a larger area from different levels of famine intensity and resulted in more deaths, making it a more appropriate natural experiment to study famine exposure and chronic diseases in middle age. Furthermore, our study extracted data from the CHARLS, a high-quality dataset of individuals over in China regarded as an opportunity to systematically assess long-term famine effects, ensuring high reliability and accuracy.

In addition to the above advantages, there are several potential limitations in our study. First, there was no clear time point for the beginning and end of the famine in China, which resulted in the ambiguity of the stage of famine exposure in the study. This may lead to misclassification bias. For instance, people born in the spring of 1959 were divided into fetal exposure groups despite the possibility that they may not have been exposed to famine during the whole fetal period. Secondly, although our study covered as many confounding factors as possible, it still lacked other important factors like dietary information, physical activity, early life socioeconomic status, among others, which may limit the accuracy of the findings. Moreover, the use of causal knowledge and graphs is expected to be applied in future studies, which could guide the selection of potential confounders. Third, although the new design of our study controlled for the effects of age, it could not adjust the effect of the different time periods between the exposure and control groups. Since different famine exposure groups were obtained from different waves, it is also difficult to adjust the clustering effect of the waves. In addition, the statistical efficiency of the model was low due to the small sample size of this study. Therefore, studies examining the association between the early life experience of famine and the risk of chronic diseases in adulthood need to be verified by large cohorts and international databases.

## Conclusions

Exposure to famine in early life increased the risk of metabolically unhealthy status in adulthood. Compared with those who did not experience famine, men exposed to famine in fetal period had a lower risk of obesity and an increased risk of MUNO in middle age. Fetal period and early childhood are the key periods to prevent metabolic abnormalities in adulthood. For the middle-aged and elderly people with normal weight who were born before or during the famine years, efforts should be made to identify different metabolic subtypes at an early stage and carry out classification, intervention and treatment.

## Data availability statement

The CHARLS databases are public and open to researchers who has applied for them. The data can be applied at: https://charls.pku.edu.cn/, further inquiries can be directed to the corresponding authors.

## Ethics statement

The studies involving human participants were reviewed and approved by the Biomedical Ethics Review Committee of Peking University. The patients/participants provided their written informed consent to participate in this study. Written informed consent was obtained from the individual(s) for the publication of any potentially identifiable images or data included in this article.

## Author contributions

PS and LA designed the study. QY and YX managed and analyzed the data. YX prepared the first draft. All authors were involved in revising the manuscript and approved the final version of the manuscript.

## Acknowledgments

All authors thank the team of the China Health and Retirement Longitudinal Study (CHARLS) for providing data and instructions of using the dataset. This research received no external funding.

## Conflict of interest

The authors declare that the research was conducted in the absence of any commercial or financial relationships that could be construed as a potential conflict of interest.

## Publisher’s note

All claims expressed in this article are solely those of the authors and do not necessarily represent those of their affiliated organizations, or those of the publisher, the editors and the reviewers. Any product that may be evaluated in this article, or claim that may be made by its manufacturer, is not guaranteed or endorsed by the publisher.
