# Supplementary material for: Chinese famine exposure in early life and metabolic obesity phenotype in middle age: Results from the China health and retirement longitudinal study
Source: Front Endocrinol (Lausanne). 2022 Sep 20;13:975824. doi: 10.3389/fendo.2022.975824 (PMC9531307; doi:10.3389/fendo.2022.975824)
Supplement: Supplementary file 1 [file DataSheet_1.docx]

Supplementary Material

**Supplementary Table S1** CSSI at province level

**Supplementary Table S2** Demographic characteristics of included and excluded participants

*Note: Data were presented as n (%) or means ± standard deviation (SD).*

*P-value represented T-test for continuous variables or χ2-test for categorical variables.*

*Abbreviations: CVD, cardiovascular disease.*

**Supplementary Table S3** The associations of the fetal-exposed group with metabolic status, obesity and metabolic obesity phenotypes compared with the age-balanced group [OR (95%CI)]

*Note: OR, odds ratio; CI, confidence interval.*

*Crude model did not adjust for any covariate. Fully adjusted model was adjusted for age, gender (except for sex-stratified analyses), marital status, education, region, smoking, drinking status, CVD history and CSSI.*

*The age-balanced group combined non-exposed group (pre-famine) and early-childhood exposed group (post-famine) from 2015 wave.*

*Abbreviations: MH, metabolically healthy; MU, metabolically unhealthy; NO, non-overweight/obesity; MHNO, metabolically healthy non-obesity; MUNO, metabolically unhealthy non-obesity; MHO, metabolically healthy obesity; MUO, metabolically unhealthy obesity.*

**Supplementary Figure S1.** Flow chart for participants included in this study

*Note: Missing covariate refers to any incompleteness information of gender, marital status, educational attainment, smoking status, drinking status. Missing outcome variable refers to any incompleteness information of metabolic obesity phenotype.*
