# Supplementary material for: Chinese famine exposure in early life and metabolic obesity phenotype in middle age: Results from the China health and retirement longitudinal study
Source: Front Endocrinol (Lausanne). 2022 Sep 20;13:975824. doi: 10.3389/fendo.2022.975824 (PMC9531307; doi:10.3389/fendo.2022.975824)
Supplement: Supplementary file 2 [file DataSheet_2.docx]

**Supplementary Table S1** CSSI at Province level

| Province | N_famine_ | N_nonfamine_ | Difference | CSSI |
| --- | --- | --- | --- | --- |
| Sichuan (SC) | 21,938 | 52,806 | 30,868 | 58.5 |
| Anhui (AH) | 11,656 | 22,007 | 10,351 | 47.0 |
| Guizhou (GZ) | 6,827 | 13,652 | 6,825 | 50.0 |
| Hunan (HuN) | 15,157 | 30,479 | 15,322 | 50.3 |
| Henan (HeN) | 24,450 | 40,468 | 16,018 | 39.6 |
| Qinghai (QH) | 1,025 | 1,587 | 562 | 35.4 |
| Jiangsu (JS) | 23,222 | 42,083 | 18,861 | 44.8 |
| Ningxia (NX) | 1,161 | 1,835 | 674 | 36.7 |
| Guangxi (GX) | 10,967 | 18,427 | 7,460 | 40.5 |
| Gansu (GS) | 6,664 | 10,243 | 3,579 | 34.9 |
| Shandong (SD) | 30,878 | 52,916 | 22,038 | 41.6 |
| Yunnan (YN) | 10,837 | 17,807 | 6,970 | 39.1 |
| Hubei (HuB) | 17,484 | 30,623 | 13,139 | 42.9 |
| Fujian (FJ) | 9,852 | 16,119 | 6,267 | 38.9 |
| Hebei (HeB) | 23,406 | 37,512 | 14,106 | 37.6 |
| Zhejiang (ZJ) | 17,087 | 27,955 | 10,868 | 38.9 |
| Jiangxi (JX) | 12,153 | 18,438 | 6,285 | 34.1 |
| Guangdong (GD) | 23,293 | 36,064 | 12,771 | 35.4 |
| Liaoning (LN) | 18,324 | 29,553 | 11,229 | 38.0 |
| Shanxi (SX) | 12,059 | 16,667 | 4,608 | 27.6 |
| Shaanxi (SaX) | 13,586 | 17,747 | 4,161 | 23.4 |
| Jilin (JL) | 11,835 | 14,983 | 3,148 | 21.0 |
| Inner Mongolia (IM) | 9,532 | 12,382 | 2,850 | 23.0 |
| Heilongjiang (HLJ) | 15,529 | 18,731 | 3,202 | 17.1 |
| Beijing (BJ) | 5,594 | 7,762 | 2,168 | 27.9 |
| Tianjin (TJ) | 4,293 | 6,554 | 2,261 | 34.5 |
| Shanghai (SH) | 7,487 | 12,173 | 4,686 | 38.5 |

*Notes: CSSI (%): (**N_nonfamine_ – N_famine_) ×100/ N_nonfamine_*

*Difference: N_nonfamine_ – N_famine_*

*Source: 1% China 2000 Census, place of birth.*

**Supplementary Table S2** Demographic characteristics of included and excluded participants

|  | | **Included** | **Excluded** | ***P*-value** |
| --- | --- | --- | --- | --- |
| Total, n | | 2684 | 4655 |  |
| Sex, n (%) | |  |  | <0.001 |
|  | Male | 1630 (60.7) | 1862 (40.2) |  |
|  | Female | 1054 (39.3) | 2767 (59.8) |  |
| Age, years | | 53.10 ± 2.14 | 52.90 ± 2.17 | <0.001 |
| Marital status, n (%) | |  |  | 0.061 |
|  | Unmarried | 134 (5.0) | 281 (6.0) |  |
|  | Married | 2550 (95.0) | 4373 (94.0) |  |
| Education, n (%) | |  |  | 0.003 |
|  | Primary school or below | 1327 (49.4) | 2136 (52.5) |  |
|  | Junior school | 829 (30.9) | 1102 (27.1) |  |
|  | Senior school or above | 528 (19.7) | 829 (20.4) |  |
| Region, n (%) | |  |  | 0.003 |
|  | Urban | 1035 (38.6) | 1959 (42.1) |  |
|  | Rural | 1649 (61.4) | 2696 (57.9) |  |
| Smoking, n (%) | |  |  | ＜0.001 |
|  | Non-smoker | 1319 (49.1) | 3050 (68.6) |  |
|  | Smoker | 1365 (50.9) | 1394 (31.4) |  |
| Drinking, n (%) | |  |  | ＜0.001 |
|  | Non-drinker | 1616 (60.2) | 3177 (69.2) |  |
|  | Drinker | 1068 (39.8) | 1417 (30.8) |  |
| CVD history, n (%) | |  |  | 0.036 |
|  | Yes | 257 (9.6) | 357 (8.1) |  |
|  | No | 2427(90.4) | 4034 (91.9) |  |

*Note: Data were presented as n (%) or means ± standard deviation (SD).*

*P-value represented T-test for continuous variables or χ2-test for categorical variables.*

*Abbreviations: CVD, cardiovascular disease.*

**Supplementary Table S3** The associations of the fetal-exposed group with metabolic status, obesity and metabolic obesity phenotypes compared with the age-balanced group [OR (95%CI)]

| **Characteristics** | | **Metabolic status** | |  | **Obesity** | |  | **Metabolic obesity phenotype** | | | |
| --- | --- | --- | --- | --- | --- | --- | --- | --- | --- | --- | --- |
|  |  | **MH** | **MU** |  | **NO** | **Overweight/obesity** |  | **MHNO** | **MUNO** | **MHO** | **MUO** |
| Overall | |  |  |  |  |  |  |  |  |  |  |
|  | Crude model | 1 | 1.02 (0.84,1.24) |  | 1 | 1.04 (0.86,1.27) |  | 1 | 0.89 (0.65,1.21) | 0.94 (0.71,1.24) | 1.04 (0.82,1.33) |
|  | Fully adjusted model | 1 | 1.02 (0.82,1.27) |  | 1 | 1.02 (0.83,1.25) |  | 1 | 0.91 (0.65,1.27) | 0.92 (0.69,1.22) | 1.03 (0.79,1.35) |
| Male | |  |  |  |  |  |  |  |  |  |  |
|  | Crude model | 1 | 0.97 (0.74,1.27) |  | 1 | 0.96 (0.75,1.23) |  | 1 | 0.87 (0.56,1.35) | 0.91 (0.66,1.24) | 0.97 (0.70,1.35) |
|  | Fully adjusted model | 1 | 0.99 (0.75,1.31) |  | 1 | 0.91 (0.70,1.18) |  | 1 | 1.01 (0.64,1.58) | 0.86 (0.62,1.19) | 0.96 (0.68,1.36) |
| Female | |  |  |  |  |  |  |  |  |  |  |
|  | Crude model | 1 | 1.09 (0.75,1.59) |  | 1 | 1.20 (0.86,1.68) |  | 1 | 0.99 (0.57,1.74) | 1.11 (0.58,2.12) | 1.21 (0.74,1.97) |
|  | Fully adjusted model | 1 | 1.06 (0.73,1.56) |  | 1 | 1.23(0.87,1.74) |  | 1 | 0.96 (0.54,1.71) | 1.20 (0.61,2.36) | 1.22 (0.74,2.00) |

*Note: OR, odds ratio; CI, confidence interval.*

*Crude model did not adjust for any covariate. Fully adjusted model was adjusted for age, gender (except for sex-stratified analyses), marital status, education, region, smoking, drinking status, CVD history and CSSI.*

*The age-balanced group combined non-exposed group (pre-famine) and early-childhood exposed group (post-famine) from 2015 wave.*

*Abbreviations: MH, metabolically healthy; MU, metabolically unhealthy; NO, non-overweight/obesity; MHNO, metabolically healthy non-obesity; MUNO, metabolically unhealthy non-obesity; MHO, metabolically healthy obesity; MUO, metabolically unhealthy obesity.*
