# Supplementary figures and images for: Chinese famine exposure in early life and metabolic obesity phenotype in middle age: Results from the China health and retirement longitudinal study
Source: Front Endocrinol (Lausanne). 2022 Sep 20;13:975824. doi: 10.3389/fendo.2022.975824 (PMC9531307; doi:10.3389/fendo.2022.975824)

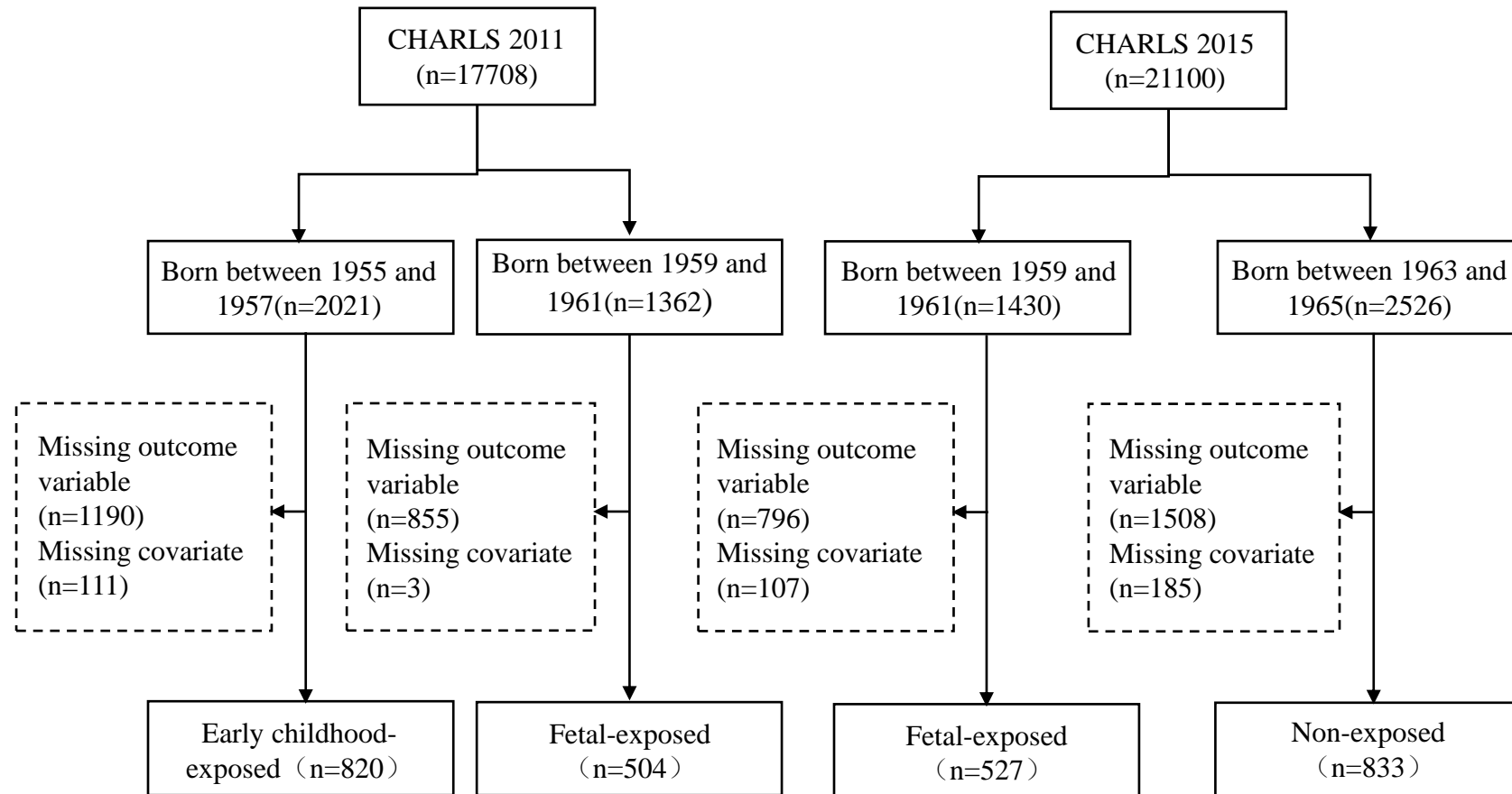

Supplement: Supplementary file 3 [file DataSheet_3.pdf]
